# Pd/Ni-metal–organic framework-derived porous carbon nanosheets for efficient CO oxidation over a wide pH range†

**DOI:** 10.1039/d2na00455k

**Published:** 2022-09-05

**Authors:** Adewale K. Ipadeola, Kamel Eid, Aboubakr M. Abdullah, Rashid S. Al-Hajri, Kenneth I. Ozoemena

**Affiliations:** Center for Advanced Materials, Qatar University Doha 2713 Qatar bakr@qu.edu.qa; Gas Processing Center (GPC), College of Engineering, Qatar University Doha 2713 Qatar kamel.eid@qu.edu.qa; Petroleum and Chemical Engineering Department, Sultan Qaboos University Muscat Oman rashidh@squ.edu.om; Molecular Sciences Institute, School of Chemistry, University of the Witwatersrand Private Bag 3, PO Wits Johannesburg 2050 South Africa Kenneth.ozoemena@wits.ac.za

## Abstract

Metal nanocrystal ornamented metal–organic frameworks (MOFs) are of particular interest in multidisciplinary applications; however, their electrocatalytic CO oxidation performance over wide pH ranges is not yet reported. Herein, Ni-MOF-derived hierarchical porous carbon nanosheets (Ni-MOF/PC) with abundant Ni–N_*x*_ sites decorated with Pd nanocrystals (Pd/Ni-MOF/PC) were synthesized by microwave-irradiation (MW-I) followed by annealing at 900 °C and subsequent etching of Ni-MOF/C prior to Pd deposition. The fabrication mechanism comprises the generation of self-reduced reducing gases from triethylamine during the annealing and selective chemical etching of Ni, thereby facilitating the reduction of Ni-anchored MOF and Pd nanocrystal deposition with the aid of ethylene glycol and MW-I to yield Pd/Ni–N_*x*_ enriched MOF/PC. The synthetic strategies endear the Pd/Ni-MOF/PC with unique physicochemical merits: abundant defects, interconnected pores, high electrical conductivity, high surface area, Ni-deficient but more active sites for Pd/Ni–N_*x*_ in porous carbon nanosheets, and synergism. These merits endowed the CO oxidation activity and stability on Pd/Ni-MOF/PC substantially than those of Pd/Ni-MOF/C and Pd/C catalysts in wide pH conditions (*i.e.*, KOH, HClO_4,_ and NaHCO_3_). The CO oxidation activity study reveals the utilization of MOF/PC with metal nanocrystals (Pd/Ni) in CO oxidation catalysis.

## Abstract 2

Ni-MOF-derived hierarchical porous carbon nanosheets (Ni-MOF/PC) decorated with Pd nanocrystals (Pd/Ni-MOF/PC) have high electrocatalytic CO oxidation activity in KOH, HClO_4_, and NaHCO_3_ electrolytes than Pd/C and Pd/Ni-MOF/C.

## Graphical abstract

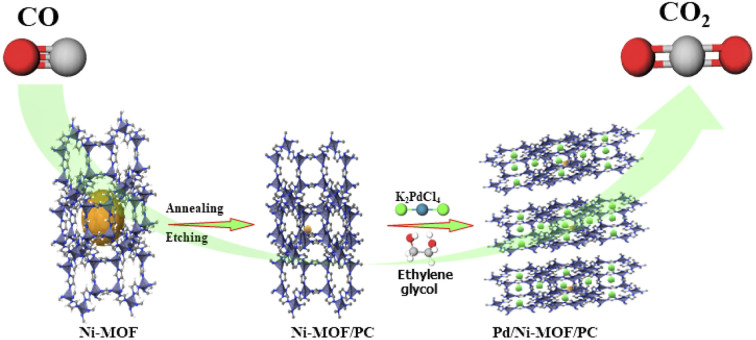

## Introduction

Alcohol-based (ethanol,^1,2^ methanol,^3,4^ glucose^5^) fuel cells are green, sustainable, and effective energy sources; however, carbon monoxide (CO) poisoning^6–8^ is among the most crucial factors precluding the large-scale applications of such devices. CO oxidation is of great importance in multi-disciplinary industrial and environmental applications. There are various methods for CO oxidation, such as thermal,^9–12^ electrochemical,^10^ and photo-electrochemical,^10,13^ driven by various catalysts. Electrochemical CO oxidation is highly preferred environmentally owing to its lower energy demand and easy operation.^14^ Noble metals, particularly Pd-based catalysts, are the most impressive electrocatalysts for CO oxidation owing to their outstanding ability to promote the adsorption/dissociation of reactants (*i.e.*, CO and O_2_) alongside the oxidation of hydrogen under low applied potentials.^14–21^ However, the ceaseless price upsurge and earth scarcity, besides instability for the long-term, are the main stumbling blocks for large-scale applications. Various efforts were dedicated to solving these barriers in tailoring the size, shape, and lowering composition of Pd nanocrystals and their supports, thereby reducing the cost of the catalyst.^14–23^ Altering the d-band center of Pd by other metals allows the self-production of reactive oxygenated species (*i.e.*, ˙OH radicals), which accelerate the CO oxidation kinetics and tolerate the adsorption of intermediates.^14–21^ For example, Pd nanodendrites enhanced the CO oxidation mass activity (58 mA mg_Pd_^−1^) by 1.9 fold than commercial Pd/C.^24^ The CO oxidation activity and durability of Pd_40_Ni_43_P_17_ outperformed the Pd/C catalyst, originating from the *in situ* formation of ˙OH radicals on Ni active sites.^25^ The CO oxidation mass activity of FePd nanocrystals (0.35 A mg_Pd_^−1^) was superior to that of Pd/C (0.18 A mg_Pd_^−1^) by 1.9 fold owing to tolerable CO_ads_ over FePd.^26^ The CO oxidation activity and stability of Pd-based catalysts are augmented using supports, particularly carbon-based materials, due to their rich electron density, high electrical conductivity, and ability to stabilize Pd nanocrystals against aggregation. For example, Pd_4_Au_1_/C boosted the CO oxidation current density by 1.86 folds than Pt/C and 1.41 folds than Pd/C owing to Au effect.^27^ Likewise, the CO oxidation activity of Pd_40_Ru_5_/graphene nanosheets (GNS) outperformed 40% Pd/GNs in KOH electrolyte.^28^ The CO oxidation mass activity of Pd_1_Sn_0.40_/TiO_2_–GO (∼1500 mA mg_Pd_^−1^) was 6.07 folds greater than that of Pt_1_Sn_0.40_/TiO_2_–GO and 8.82 folds than that of Pd_1_Sn_0.40_/C due to the presence of co-supports that enhance the CO_ads_.^29^

MOFs are new classes of materials and their carbonized derivatives have unique physiochemical properties of carbon and catalytic merits of metals (*i.e.*, high surface areas, multiple unsaturated coordination sites, well-crystalline structures, abundant active sites, and interior/exterior cavities), which can provide interconnected channels for reactants during CO oxidation.^30–42^ Moreover, MOFs with numerous unsaturated coordination sites and open inner/outer cavities can accommodate Pd nanocrystals, which results in higher stability aggregation and maximize Pd utilization during CO oxidation.^38,43–47^ There are a few reports on using MOFs and their derivatives as supports for Pd nanocrystals for thermal CO oxidation.^38,43–48^ However, the electrochemical CO oxidation performance of MOF-based catalysts with or without Pd nanocrystals is not yet reported to the best of our knowledge.^38,43–47^

In pursuit of this aim, we rationally synthesized Pd/Ni-MOF/PC for electrocatalytic CO oxidation in different electrolytes. The synthesis approach is simple and green, comprising microwave irradiation, annealing, and etching of the sacrificial template (Ni-MOF/C) before Pd dispersion to form Pd/Ni-MOF/PC with porous carbon nanosheets and abundant Pd/Ni–N_*x*_ active sites. Unlike traditional preparation methods (*i.e.*, seed-mediated, solvothermal, and chemical reduction), microwave-irradiation is energy-efficient, environmentally benign, and provides a uniform heating mechanism utilizing dipolar polarization and ionic conduction, resulting in prompt nucleation and growth of small-sized Pd nanoparticles uniformly distributed over Ni-MOF/PC without the need for reducing agents or multiple reaction steps.^49–53^ The partially etched Ni-MOF/C resulted in more exposed Ni–N_*x*_ active sites and porous C in Pd/Ni-MOF/PC. The electrocatalytic CO oxidation performances of Pd/Ni-MOF/PC and without etching (Pd/Ni-MOF/C) are benchmarked with commercial Pd/C catalysts in KOH, HClO_4_, and NaHCO_3_.

## Materials and methods

### Materials

Nickel(ii) nitrate hexahydrate (Ni(NO_3_)_2_·6H_2_O, 94.5%), potassium palladium(ii) chloride (K_2_PdCl_4,_ 98%), trimesic acid (C_9_H_6_O_6_, 95%), ethylene glycol ((EG), 99.8%), triethylamine (>99.5%), dimethylformamide (DMF, 99.8%), HClO_4_, NaHCO_3_ and KOH (>98%), commercial Pt/C (20 wt% Pt) and Pd/C catalyst (20 wt%) were purchased from Sigma-Aldrich Chemie GmbH (Munich, Germany).

### Preparation of Ni-MOF-derived porous carbon (Ni-MOF/PC)

Ni-MOF/PC was synthesized by mixing Ni(NO_3_)_2_·6H_2_O (0.44 g) and (0.36 g) of trimesic acid (0.36 g) in a mixture of triethylamine (1.5 mL) and DMF (50 mL) under stirring at 25 °C. Then, the solution was poured into a Teflon container and subjected to microwave irradiation (at 600 W, 30 min). The precipitates obtained were washed and dried in a vacuum oven at 60 °C, followed by annealing at 900 °C for 4 h (5° min^−1^ ramping rate) to yield Ni-MOF/C. The obtained powder was soaked in an aqueous solution of HCl (3 M) for 48 h to form Ni-deficient-MOF with enriched porous carbon nanosheets (Ni-MOF/PC).

### Preparation of Pd/Ni-MOF/PC and Pd/Ni-MOF/C

Pd/Ni-MOF/PC and Pd/Ni-MOF/C were synthesized by mixing K_2_PdCl_4_ (61.35 mg) with EG (50 mL) under stirring at 25 °C for 30 min, and the pH was adjusted to 12 by KOH (1 M). Then, Ni-MOF/PC and Ni-MOF/C, respectively, were added. The solutions were placed in the microwave for irradiation at 600 W for 1 h. An aqueous solution of HCl (3 M) was used to lower the resulting solutions' pH (3), forming precipitates. The precipitates were washed with ethanol and H_2_O and then dried at 80 °C for 24 h under a vacuum.

### Materials characterization

The morphology and composition of the electrocatalysts were analyzed by a scanning electron microscope ((SEM), Hitachi S-4800, Hitachi, Tokyo, Japan) equipped with an energy dispersive X-ray analyzer (EDX) and a transmission electron microscope ((TEM), TecnaiG220, FEI, Hillsboro, OR, USA). Their electronic structures and surface compositions were analyzed using X-ray photoelectron spectroscopy ((XPS), Ultra DLD XPS Kratos, Manchester, UK). The crystallinity was investigated by X-ray diffraction (XRD, X'Pert-Pro MPD, PANalytical Co., Almelo, Netherlands). Raman spectra were recorded on a Raman instrument (Thermo Scientific) under 532 nm laser excitation. Inductively coupled plasma optical emission spectrometry (ICP-OES, Agilent 5800 DV) was used to estimate the catalyst loading on working electrodes. Brunauer–Emmett–Teller (BET) surface area analysis (Horiba SA-9600) was performed to determine the specific surface area, pore volume, and pore sizes.

### CO oxidation reaction

The electrochemical CO oxidation was carried out on a Gamry potentiostat (Reference 3000, Gamry Co., Warminster, PA, USA) using the three-electrode cell involving Pt wire, Ag/AgCl, and glassy carbon (GC), as counter, reference, and the working electrodes, respectively. 2 mg of each catalyst was dispersed in an aqueous solution of isopropanol/H_2_O (3/1 v/v ratio) and drop-casted onto the GC electrodes, 3 μL of Nafion solution (0.05 wt%) was added, and electrodes were left to dry in an oven under vacuum at 80 °C for 1 h. The loading of Pd in the electrocatalysts on the GCE was approximately 0.020 ± 0.002 mg_Pd_ cm^−2^, as determined by ICP-OES. The electrochemical active surface area (ECSA) was calculated by the integration of hydrogen under-potential adsorption/desorption (HUPD) from CV curves. All the potentials were calibrated to a reversible hydrogen electrode (RHE), according to the equation: *E*(RHE) = *E*(Ag/AgCl) + 0.197*V* + 0.059 × pH.

## Results and discussion

**Fig. 1:**
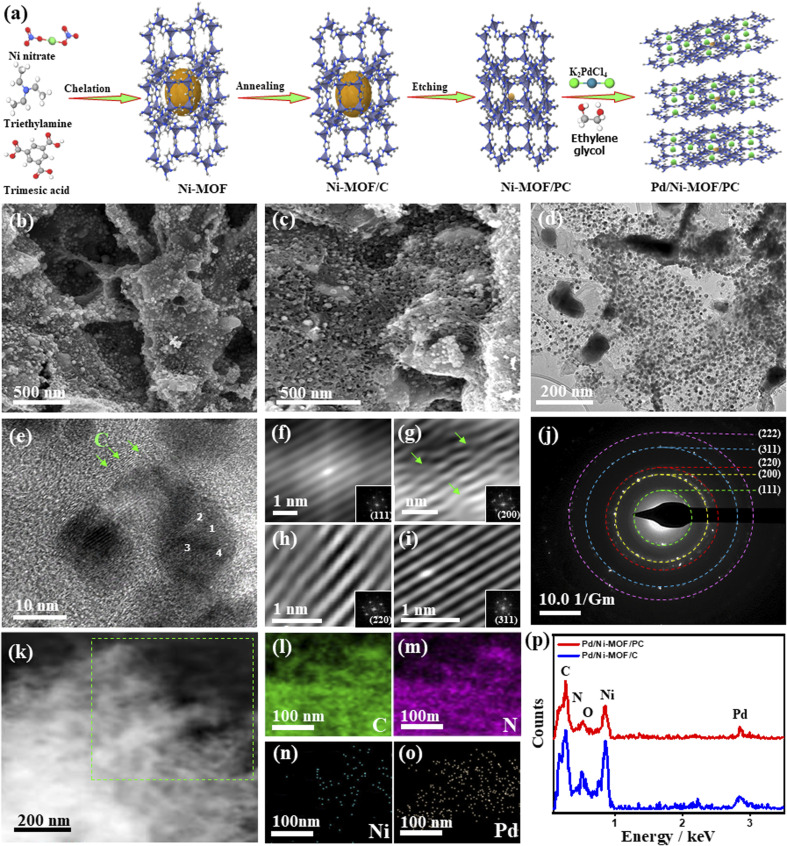
(a) The preparation process of Pd/Ni-MOF/PC. (b) SEM image of Pd/Ni-MOF/PC and its (c) SEM image, (d) TEM image, (e) HRTEM image, (f–i) Fourier filtered HRTEM images of the numbered areas (1–4) in (e), respectively, (j) SAED, (k), HADDF-STEM image of Pd/Ni-MOF/PC and its (l–o) elemental mapping analysis of the marked area in (k). (p) EDX analysis of Pd/Ni-MOF/PC and Pd/Ni-MOF/PC.

Fig. 1a shows the synthesis process of Pd/Ni-MOF/PC as outlined: the Ni-MOF template prepared by microwave irradiation was subsequently annealed under N_2_ to afford abundant Ni nanocrystals anchored on the N_*x*_-sites of MOF/C with the assistance of the self-reduced gases (*i.e.*, NH_3_, C_*x*_H_*y*_, and H_2_) emitted from triethylamine at a high carbonization temperature. Then, the selective chemical etching of Ni-MOF/C in HCl allows the partial removal of Ni nanocrystals to produce porous carbon nanosheets with abundant defects and cavities (*i.e.*, Ni-MOF/PC) due to the instability of Ni in acidic solutions. The microwave-illumination of Ni-MOF/PC and Ni-MOF/C in an ethylene glycol solution containing Pd produced Pd nanocrystal-ornamented Ni-MOF/PC (Pd/Ni-MOF/PC) and Pd/Ni-MOF/C, respectively, owing to the reduction and microwave absorption power of ethylene glycol.^22,54^Fig. 1b shows the SEM image of Pd/Ni-MOF/C with a stacked sheet-like morphology without any obvious porosity, and the TEM image reveals the good distribution of Pd/Ni nanocrystals in a spherical-like shape over carbon nanosheets (Fig. S1a†).

The average diameter of Pd nanocrystals is about 10.8 nm (Fig. S1b†). The TEM image of Pd/Ni-MOF/C displays the lattice fringes of Pd nanocrystals without any crystalline defects with an interlayer spacing of 0.22 nm assigned to the {111} facet of face-centered cubic (fcc) Pd in addition to the amorphous–crystalline structure of carbon as indicated by the arrows in Fig. S1c.† The selected area electron diffraction (SAED) patterns imply the typical diffraction rings ascribed to the {111}, {200}, {220}, {311}, and {222} crystal planes of fcc Pd (Fig. S1d†), as usually observed for Pd nanocrystals. The etching of Ni-MOF/C by HCl in Pd/Ni-MOF/PC yields well-defined hierarchical porous nanosheets with interconnected pores and diameters ranging from 5 to 50 nm due to the partial etching of Ni nanocrystals (Fig. 1c). The interconnected pores are important for facilitating CO absorption and migration along with maximizing the utilization of buried Pd and Ni during CO oxidation. The TEM image of Pd/Ni-MOF/PC displays the uniform distribution of Pd nanocrystals over porous carbon nanosheets (Fig. 1d). The average size of thus formed Pd nanocrystals in Pd/Ni-MOF/PC is 7.5 nm, which decreased significantly relative to Pd/Ni-MOF/C, owing to the etching effect and instability of Ni nanocrystals in acidic solutions (Fig. S2†).^55,56^ The HRTEM image of Pd/Ni-MOF/PC reveals the lattice fringes of Pd nanocrystals and amorphous–crystalline structure of carbon, as indicated by the arrows (Fig. 1e). The Fourier-filtered HRTEM images of Pd nanocrystals demonstrate the lattice fringes with multiple crystalline defects, such as interfacial dislocation, intragranular dislocation, and stacking fault, as indicated by the arrows (Fig. 1f–i). The estimated interplanar spacings 2.236, 1.936, 1.369, 1.170, and 1.116 Å are assigned to the {111}, {200}, {220}, {311}, and {222} facets of face-centered-cubic (fcc) of Pd, respectively, as usually observed in Pd-based catalysts.^57^ This is in line with the SAED patterns, which reveal the diffraction rings ascribed to fcc Pd (Fig. 1j). The HAADF-STEM of Pd/Ni-MOF/PC discloses the formation of hierarchical porous nanosheets (Fig. 1k). The element mapping analysis validates the presence of C, N, Ni, and Pd in Pd/Ni-MOF/PC with atomic contents of 54.12, 8.02, 15.08, and 22.15%, respectively (Fig. 1l–o), as further proved by the EDX analysis (Fig. 1p). The EDX analysis of Pd/Ni-MOF/C depicts the co-existence of Pd, Ni, C, O, and N with the atomic contents of 10.29, 20.80, 47.86, 10.79, and 10.44% in Pd/Ni-MOF/C, respectively (Fig. 1p). The presence of O in Pd/Ni-MOF/C is plausibly due to the instability of Ni in air resulting in the formation of passivating oxide layers over Ni, which disappear after etching and possibly allow Pd–NiN_*x*_ coordination in Pd/Ni-MOF/PC.

**Fig. 2:**
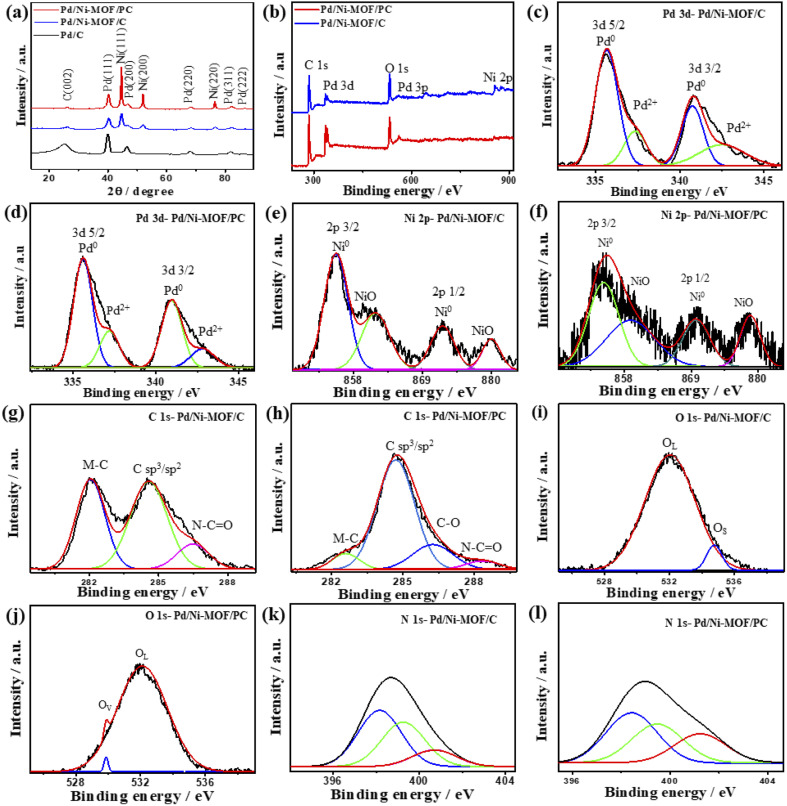
(a) XRD, (b) XPS survey, high-resolution XPS spectra of (c and d) Pd 3d, (e and f) Ni 2p, (g and h) C 1s, (i and j) O 1s and (k and l) N 1s of Pd/Ni-MOF/C and Pd/Ni-MOF/PC, respectively.

Notably, in the absence of microwave irradiation, we could not obtain Pd nanoparticles, implying the significant effect of microwave irradiation to allow the reduction of the Pd precursor and form Pd nanoparticles without the use of a reducing agent. This is plausibly due to the uniform heating mechanism, dipolar polarization, and ionic conduction effect of microwave irradiation. The XRD of Pd/Ni-MOF/PC and Pd/Ni-MOF/C shows the diffraction patterns assigned to the (111), (200), (220), (311), and (222) facets of fcc Pd besides (111), (200) and (220) of fcc Ni, and (002) facet of graphitic C (Fig. 2a). The Pd(111) and Ni(111) facets dominate in both Pd/Ni-MOF/PC and Pd/Ni-MOF/C. The presence of the XRD diffraction patterns of Ni plausibly indicates the formation of Ni nanocrystals due to the *in situ* reducing gases generated from triethyleneamine during microwave irradiation and annealing.^48^ The diffraction patterns of Pd/Ni-MOF/PC are broadened with lower intensity relative to Pd/Ni-MOF/C due to crystals defects and the Pd/Ni–N_*x*_–C ligand effect. That is also evidenced in the slight positive shift in the 2*θ* angle value of Pd/Ni-MOF/PC compared to Pd/Ni-MOF/C and commercial Pd/C. The Pd/Ni–N_*x*_–C ligand effect in Pd/Ni-MOF/PC leads to a decrease in the Pd–Pd interatomic distance, as seen in the lower lattice parameter value (*a*) of Pd/Ni-MOF/PC (0.27 nm) than Pd/Ni-MOF/C (0.29 nm) and Pd/C (0.31 nm), serving as an evidence of higher defects in Pd/Ni-MOF/PC. The average crystallite size of Pd/Ni-MOF/PC (4.0 nm) is lower than that of Pd/Ni-MOF/C (7 nm) and Pd/C (8 nm) as obtained from the (111) peak using Scherrer's equation.

The XPS full-scan of Pd/Ni-MOF/PC and Pd/Ni-MOF/C implies the presence of Pd 3d, Ni 2p, C 1s, N 1s and O 1s (Fig. 2b). The deconvolution of Pd 3d spectra in both Pd/Ni-MOF/PC and Pd/Ni-MOF/C shows Pd^0^ 3d_5/2_ and Pd^0^ 3d_3/2_ as the main metallic phase in addition to the small peaks of PdO (Pd^2+^ 3d_5/2_ and Pd^2+^ 3d_3/2_), owing to the high reduction feature of ethylene glycol under microwave illumination (Fig. 2c and d). The fitting of Ni 2p spectra (Fig. 2e and f) reveals intense peaks for Ni^0^ 2p_3/2_ and Ni^0^ 2p_1/2_ as the main phase and NiO peak as a minor phase, but the ratio of the Ni metallic phase to the NiO phase in Pd/Ni-MOF/PC is higher than in Pd/Ni-MOF/C due to the etching effect. The presence of Ni in the metallic phase is in line with the XRD results that further infer the formation of Ni nanocrystals in Pd/Ni-MOF/PC but Ni and NiO nanocrystals in Pd/Ni-MOF/C. The C 1s spectra comprise two intensive peaks for C-bonded to metal (M–C), sp^2^/sp^3^ of C–C, and a small shoulder for N–C=O in Pd/Ni-MOF/C (Fig. 2g), but a strong peak for C–C, low M–C and N–C=O were present in Pd/Ni-MOF/PC (Fig. 2h). The O 1s spectra show lattice O (O_L_) and surface O (O_S_) in Pd/Ni-MOF/C (Fig. 2i), but O_L_ and O vacancy (O_V_) in Pd/Ni-MOF/PC (Fig. 2j). The O_V_ in Pd/Ni-MOF/PC implies the removal of a passivating oxide layer and generation of defects by HCl etchant. The N 1s spectra display pyridinic N (398.1 eV), metal–N species (399.2 eV), pyrrolic N (401.2 eV), and graphitic N (402.2 eV) in both Pd/Ni-MOF/C (Fig. 2k) and Pd/Ni-MOF/PC (Fig. 2l). The presence of metal–N species in both Pd/Ni-MOF/C and Pd/Ni-MOF/PC supports the atomic dispersion of Ni–N_*x*_ and its coordination with Pd.

The Raman spectra of Pd/Ni-MOF/PC and Pd/Ni-MOF/C show a graphitic (G) band at ∼1591 cm^−1^ assigned to the crystalline graphite sp^2^ carbon basal plane and a defective (D) band of sp^3^ carbon at ∼1333 cm^−1^ (Fig. S3a†). The broadened and decreased intensity of D and G peaks of Pd/Ni-MOF/PC relative to Pd/Ni-MOF/C are due to the etching effect, which generates various structural defects. That is additionally seen in the higher ratio of (*I*_D_/*I*_G_) of Pd/Ni-MOF/PC (0.98) than Pd/Ni-MOF/C (0.86). The structural defects of Pd/Ni-MOF/PC could act as active sites for boosting the adsorption of reactant molecules during CO oxidation.

The FTIR analysis is accrued to get more insights into the chemical nature of Pd/Ni-MOF/PC and Pd/Ni-MOF/C, which both show strong absorption peaks of the stretching mode of C=C, C–N heterocycles, and N–H (Fig. S3b†). The broadening of the N–H peak could serve as an indication for Ni–N_*x*_ coordination in both Pd/Ni-MOF/PC and Pd/Ni-MOF/C. Noticeably, broader Pd/Ni-MOF/PC peaks with less intensity compared to its counterpart (Pd/Ni-MOF/C) are ascribable to the etching effect.^22^

The pore size, pore volume, and surface area of electrocatalysts are investigated with BET analysis, where Pd/Ni-MOF/PC has a high pore size (7.60 nm), pore volume (0.1240 cm^3^ g^−1^), and surface area (153.0463 m^2^ g^−1^) than Pd/Ni-MOF/C (6.15 nm, 0.1049 cm^3^ g^−1^, and 142.2231 m^2^ g^−1^) (Table S1†). Also, well-distributed pore size and pore volume for the adsorption of gas is evidenced by the hierarchical pores of Pd/Ni-MOF/PC (Fig. S3c and d†). These results corroborate more interconnected pores and surface area of Pd/Ni-MOF/PC for the easy diffusion of intermediates or gases during CO oxidation electrocatalysis. ICP-OES was utilized to determine the metal contents in the electrocatalysts. Pd/Ni-MOF/PC has Pd (14.26 ± 0.25 wt%) and Ni (14.40 ± 0.69 wt%), while Pd/Ni-MOF/C has Pd (21.40 ± 1.05 wt%) and Ni (43.12 ± 0.91 wt%). The results show that although Pd/Ni-MOF/PC has lower metal contents than Pd/Ni-MOF/C, the etching tuned its physicochemical properties for boosted CO oxidation.

**Fig. 3:**
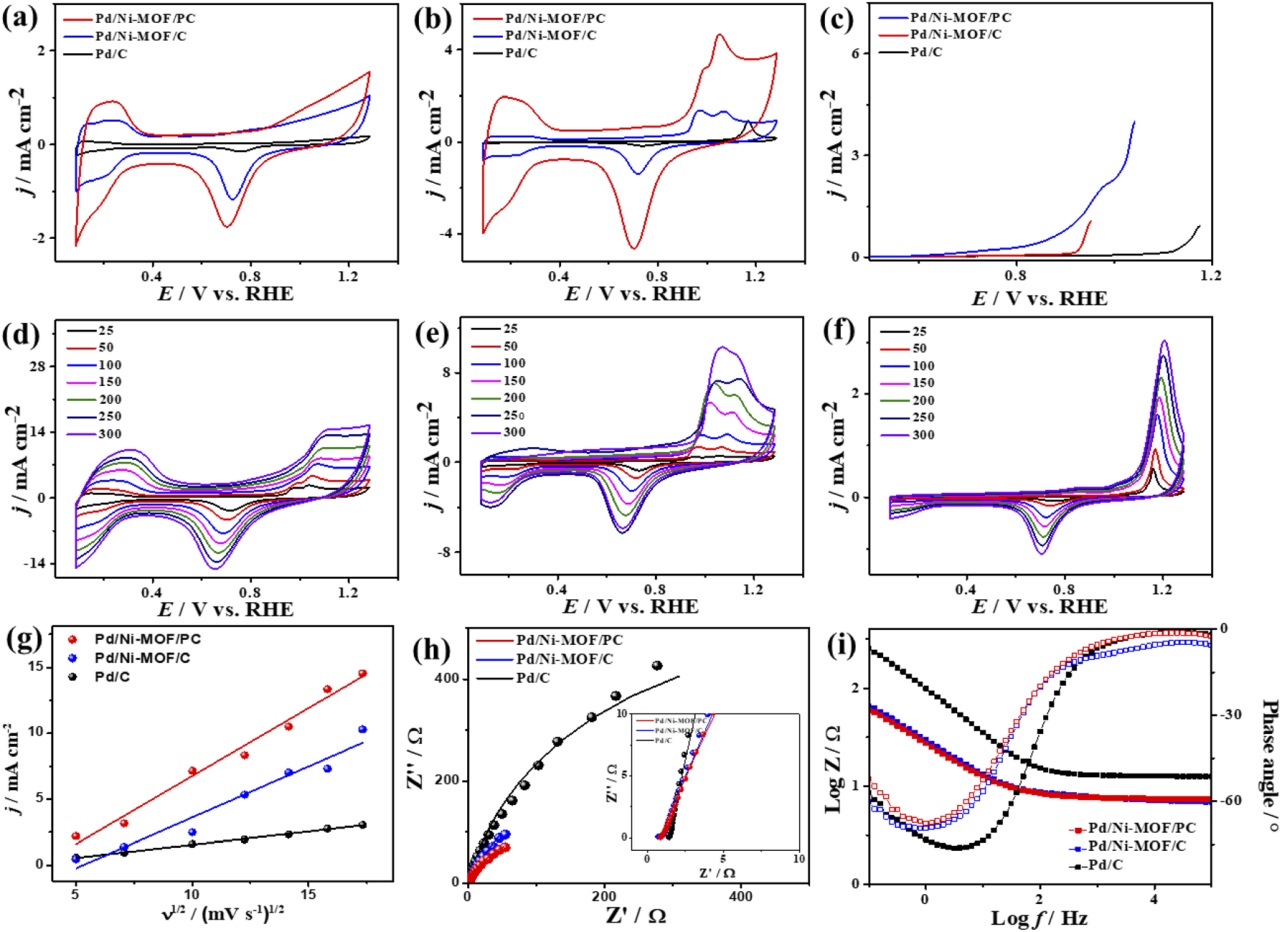
(a) CV in 0.1 M HClO_4_, (b) CV in CO-purged 0.1 M HClO_4_, (c) LSV in CO-saturated 0.1 M HClO_4_ of Pd/Ni-MOF–PC, Pd/Ni-MOF–C, and commercial Pd/C. CV measured in CO-saturated 0.1 M HClO_4_ at different scan rates of (d) Pd/Ni-MOF/C, (e) Pd/Ni-MOF/PC, (f) Pd/C, and (g) their related plots of current density *vs.* square root of scan rates. (h) Nyquist plots and (i) Bode plots measured in CO-saturated 0.1 M HClO_4_ electrolyte of Pd/Ni-MOF/PC, Pd/Ni-MOF/C, and Pd/C.

The electrochemical CO oxidation activity of Pd/Ni-MOF/PC and Pd/Ni-MOF/C is benchmarked to commercial Pd/C catalysts in different electrolytes. Fig. 3a shows the cyclic voltammograms (CV) of Pd/Ni-MOF/PC, Pd/Ni-MOF/C, and Pd/C, measured in the N_2_-saturated HClO_4_ electrolyte, which showed the voltammogram features of Pd electrocatalysts. This includes the double-layered hydrogen-adsorption/desorption (HUPD) between 0.36 V and 0.50 V and Pd–H between 0.09 V and 0.33 V, and Pd–O reduction between 0.53 V and 0.91 V without any significant peaks for Pd–O or Ni–O, which imply their stability against oxidation. Notably, the HUPD of Pd/Ni-MOF/PC is significantly higher than that of Pd/Ni-MOF/C, indicating its higher active sites and ECSA. Thereby, the ECSA of Pd/Ni-MOF/PC showed higher ECSA (69 m^2^ g^−1^), which is 2.3 times of Pd/Ni-MOF/C (30 m^2^ g^−1^) and 30 times of Pd/C (2 m^2^ g^−1^), due to the porosity, structural defects, and enhanced active sites of Pd/Ni-MOF/PC. The CV curves measured in the CO-purged HClO_4_ electrolyte of Pd/Ni-MOF/PC, Pd/Ni-MOF/C, and Pd/C show the CO-oxidation voltammogram features, including a sharp anodic oxidation current (*I*_anode_) in the positive potential region (*i.e.*, 0.81 to 1.21 V *vs.* RHE), and reduction current (*I*_reduc._) in the negative potential region along with HUPD. Both Pd/Ni-MOF/PC and Pd/Ni-MOF/C infer two noticeable *I*_anode_ peaks, plausibly owing to the Pd–Ni–N_*x*_ ligand effect. The CO oxidation activity of Pd/Ni-MOF/PC is superior to that of Pd/Ni-MOF/C and Pd/C (Fig. 3b). The *I*_anode_ of Pd/Ni-MOF/PC (4.71 mA cm^−2^) is 3.4 folds higher than that of Pd/Ni-MOF/C (1.38 mA cm^−2^) and 5.0 folds that of Pd/C (0.95 mA cm^−2^). This is due to the porous morphology, which maximizes the utilization of Pd–Ni–N_*x*_ in Pd/Ni-MOF/PC during the CO oxidation reaction. Intriguingly, the *I*_anode_ of Pd/Ni-MOF/PC (4.71 mA cm^−2^) is superior to that of previously reported PtRu, Pt/SnO_*x*_, Pd/Ti_3_C_2_T_*x*_, and PtBi nanosponge measured under similar conditions (Table S2†).^6,58–60^ The CO oxidation potential (*E*_oxi_) of Pd/Ni-MOF/PC (1.05 V) is lower than that of Pd/Ni-MOF/C (1.06 V) by 0.01 V and Pd/C (1.17 V) by 0.012 V, implying fast CO oxidation kinetics on Pd/Ni-MOF/PC (Fig. S4a†). That is further seen in the lower onset potential (*E*_onset_) of Pd/Ni-MOF/PC (0.84 V) than those of Pd/Ni-MOF/C (0.88 V) and Pd/C (1.10 V). The linear sweep voltammogram (LSV) also shows better CO oxidation kinetics on Pd/Ni-MOF/PC, as evidenced in its ability to deliver a higher *I*_anode_ than Pd/Ni-MOF/C and Pd/C under an applied potential (Fig. 3c), owing to the greater defects, higher electrical conductivity, and synergism of Pd–Ni–N_*x*_ in Pd/Ni-MOF/PC. The CVs measured at various sweeping rates from 25 to 300 mV s^−1^ reveal the progressive rise of the *I*_anode_ with increasing scan rates (*ν*) on all measured catalysts (Fig. 3d–f).

**Fig. 4:**
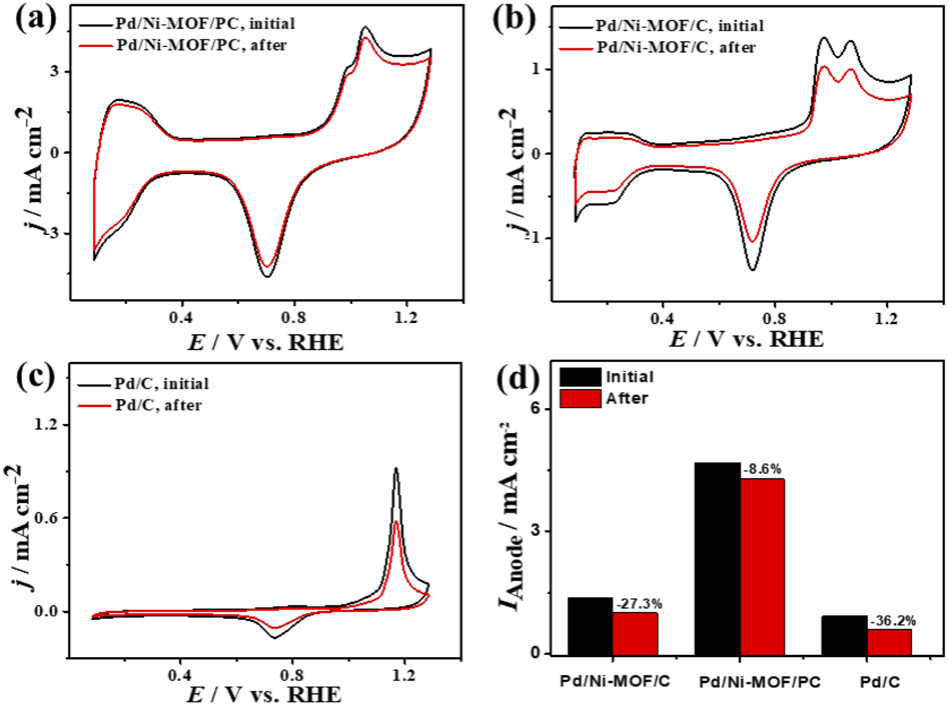
(a–c) CV stability of CO oxidation and (d) *I*_anode_ stability of Pd/Ni-MOF/PC, Pd/Ni-MOF/C, and Pd/C in CO-saturated 0.1 M HClO_4_ electrolyte.

Plotting *I*_anode_*vs. ν*^1/2^ shows a linear relationship that infers a diffusion-controlled process of CO oxidation on all catalysts, but Pd/Ni-MOF/PC displays a greater magnitude of slope (*i.e.*, 1.03) than Pd/Ni-MOF/C (0.77) and Pd/C (0.20) (Fig. 3g), serving as evidence for quicker ionic diffusion on Pd/Ni-MOF/PC. The chronoamperometry (CA) tests measured for 1200 s show the greater stability of Pd/Ni-MOF/PC than Pd/Ni-MOF/C and Pd/C catalysts, as designated by its higher current retention (Fig. S4b†). This is also shown in the CVs measured after CA tests, which unveil the same CO oxidation voltammogram features on all catalysts but with superior activity and durability on Pd/Ni-MOF/PC than Pd/Ni-MOF/C and Pd/C (Fig. 4a–c). The Pd/Ni-MOF/PC maintained 19.4% of its *I*_anode_ relative to Pd/Ni-MOF/C (72.7%) and Pd/C (63.8%) (Fig. 4d), demonstrating the significant CO oxidation stability of Pd/Ni-MOF/PC. The TEM image measured after stability tests infers the porous 2D nanosheets ornamented with Pd nanocrystals without apparent aggregation or leaching, implying structural durability (Fig. S5†).

The interfacial interaction of the electrocatalysts with CO-saturated HClO_4_ electrolytes was probed using electrochemical impedance spectroscopy (EIS), which shows a semicircle shape but with a lower diameter on Pd/Ni-MOF/PC than Pd/Ni-MOF/C and Pd/C, implying a better charge transfer across the electrolyte–electrode interface on Pd/Ni-MOF/PC (Fig. 3h).^10^ That is evidenced in fitting the Nyquist plots using the Voigt electrical equivalent circuit (Fig. S4c†) to estimate the electrolyte resistance (*R*_s_), charge transfer resistance (*R*_ct_), and constant phase element (CPE) (Table 1). Pd/Ni-MOF/PC has the lowest *R*_s_ and *R*_ct_ than Pd/Ni-MOF/C and Pd/C, indicating the quickest charge transfer across the electrolyte/electrode and lower charge mobility resistance on Pd/Ni-MOF/PC, which enhanced the CO oxidation. To get more insights into the CO oxidation process, we use the CPE with deviation (*a*) from an ideal double-layer capacitor (*C*_dl_) and Bode plots (Fig. 3i),^61^ which reveals an ideality factor of (0.5 < *a* < 1), and a lower charge mobility resistance on Pd/Ni-MOF/PC than Pd/Ni-MOF/C and Pd/C. This indicates that CO oxidation is a diffusion-controlled process coupled with the adsorption process on Pd/Ni-MOF/PC, Pd/Ni-MOF/C, and Pd/C. The phase angle in the range of 67–80° for all the catalysts further verifies that CO oxidation occurs *via* CO adsorption and diffusion-controlled processes.^62^

**Table 1:** EIS data for catalysts in CO-purged 0.1 M HClO_4_ electrolyte

	*R*_s_/Ω	*R*_ct_/Ω	CPE/μS s^*a*^	*a*
Pd/C	1.42 ± 0.22	272.70 ± 5.43	96.99 ± 1.04	0.79
Pd/Ni-MOF/C	0.89 ± 0.17	61.27 ± 1.13	359.20 ± 3.45	0.87
Pd/Ni-MOF/PC	0.83 ± 0.20	58.25 ± 0.51	228.20 ± 2.50	0.85

Thus, the CO oxidation mechanism on Pd/Ni-MOF/PC probably follows a Langmuir–Hinshelwood type reaction (eqn (i)–(iii)). This process includes the initial adsorption of CO (CO_ads_) on Pd/Ni-MOF/PC to form (CO_ads_/Pd/Ni-MOF/PC), and simultaneously, CO_ads_-free Pd nanocrystals promote H_2_ oxidation to produce OH adsorbed on Pd/Ni-MOF/PC (OH_ads_/Pd/Ni-MOF/PC).^63^ Consequently, CO_2_ is formed by the oxidation of CO_ads_ by OH_ads_ with the assistance of the oxophilic effect of Ni–N_*x*_ and then desorbed from the surface of Pd/Ni-MOF/PC. Owing to the porosity of Pd/Ni-MOF/PC, its active sites are more accessible for the reactants, and induced quick generation of OH species with the assistance of Ni–N_*x*_ promotes the CO oxidation activity.^63^

i H_2_O + CO + 2M* ↔ M*–CO_ads_ + M*–OH_ads_ + H^+^ + e^−^

ii M*–CO_ads_ + M*–OH_ads_ → 2M*–[CO–OH]_ads_

iii M*–[CO–OH]_ads_ ↔ CO_2_ + H^+^ + M* + e^−^

M* is the active site “Pd–Ni–N_*x*_” of the catalyst, and ads is the adsorbed species.

**Fig. 5:**
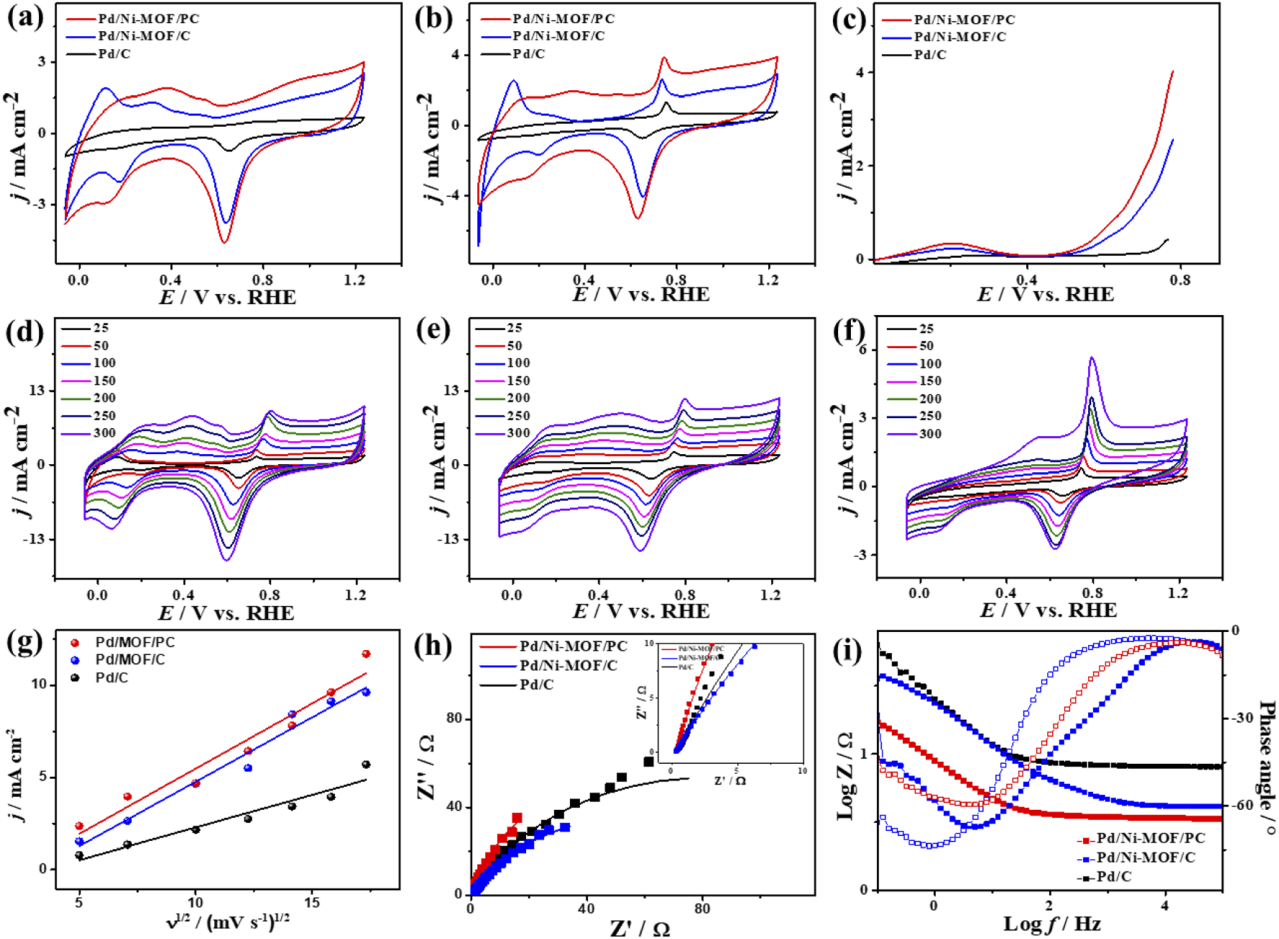
(a) CV in 0.1 M KOH, (b) CV in CO-saturated 0.1 M KOH, and (c) LSV in CO-saturated 0.1 M KOH of Pd/Ni-MOF–PC, Pd/Ni-MOF–C, and commercial Pd/C. CV measured in CO-saturated 0.1 M KOH at different scan rates of (d) Pd/Ni-MOF/PC, (e) Pd/Ni-MOF/C, and (f) Pd/C and their (g) related plots of current density *vs.* square root of scan rates, (h) Nyquist plots and (i) Bode plots measured in CO-saturated 0.1 M KOH electrolyte of Pd/Ni-MOF/PC, Pd/Ni-MOF/C and Pd/C.

The CV curves of the catalysts measured in KOH electrolyte only show the ideal voltammogram features of Pd-based catalysts but with a greater HUPD of Pd/Ni-MOF/PC and Pd/Ni-MOF/C than that of Pd/C, originating from their higher ECSA (Fig. 5a). The ECSA of Pd/Ni-MOF/PC (87.0 m^2^ g^−1^) was 1.56 and 6.59 times higher than that of Pd/Ni-MOF/C (55.7 m^2^ g^−1^) and Pd/C (13.2 m^2^ g^−1^). The CVs measured in CO-saturated KOH reveal the CO oxidation voltammogram with an apparent *I*_anode_ and *I*_cathode_ but with a grander activity of Pd/Ni-MOF/PC. The *I*_anode_ of Pd/Ni-MOF/PC (3.94 mA cm^−2^) is greater than that of Pd/Ni-MOF/C (2.65 mA cm^−2^) and Pd/C (1.37 mA cm^−2^) by 1.48 and 2.87 times, respectively. This implies the maximized utilization of Pd–Ni–N_*x*_ during CO oxidation on Pd/Ni-MOF/PC due to the nanosheet morphology with interconnected pores. That is seen in the higher *I*_anode_ of Pd/Ni-MOF/PC than that of Pd/Ni-MOF/C and Pd/C under any applied potential, implying the enhanced CO oxidation kinetics on Pd/Ni-MOF/PC (Fig. 5b and c). That is additionally seen in the earlier *E*_onset_/*E*_oxi_ of Pd/Ni-MOF/PC (0.65 V/0.74 V) relative to Pd/Ni-MOF/C (0.53 V/0.73 V) and Pd/C (0.70 V/0.75 V) (Fig. S6a†). The *I*_anode_ increased steadily with increasing *ν* from 25 to 300 mV s^−1^ on the as-synthesized catalysts but with higher *I*_anode_ values on Pd/Ni-MOF/PC than Pd/Ni-MOF/C and Pd/C (Fig. 5d–f). The linear relationship between *I*_anodic_ and *ν*^1/2^ suggests the diffusion-controlled process of CO oxidation. However, Pd/Ni-MOF/PC reveals a larger line slope of (0.71) than Pd/Ni-MOF/C (0.70) and Pd/C (0.35) (Fig. 5g). That serves as a proof of the better charge mobility kinetics on Pd/Ni-MOF/PC, as proved by the EIS.^10^ The Nyquist plots of EIS display semicircle curves but Pd/Ni-MOF/PC shows a significantly lower semicircle diameter than Pd/Ni-MOF/PC and Pd/C (Fig. 5h), implying a better electrolyte–electrode interaction and quicker charge mobility on Pd/Ni-MOF/PC. That is seen in the lower *R*_s_ and *R*_ct_ besides a greater CPE on Pd/Ni-MOF/PC relative to Pd/Ni-MOF/C and Pd/C (Table 2).

**Table 2:** EIS data for catalysts in CO-purged 0.1 M KOH electrolyte

	*R*_s_/Ω	*R*_ct_/Ω	CPE/μS s^*a*^	*a*
Pd/C	0.35 ± 0.02	62.32 ± 0.43	133.53 ± 3.72	0.79
Pd/Ni-MOF/C	0.34 ± 0.07	34.65 ± 0.31	409.09 ± 5.14	0.91
Pd/Ni-MOF/PC	0.33 ± 0.02	16.99 ± 0.48	513.04 ± 5.31	0.89

**Fig. 6:**
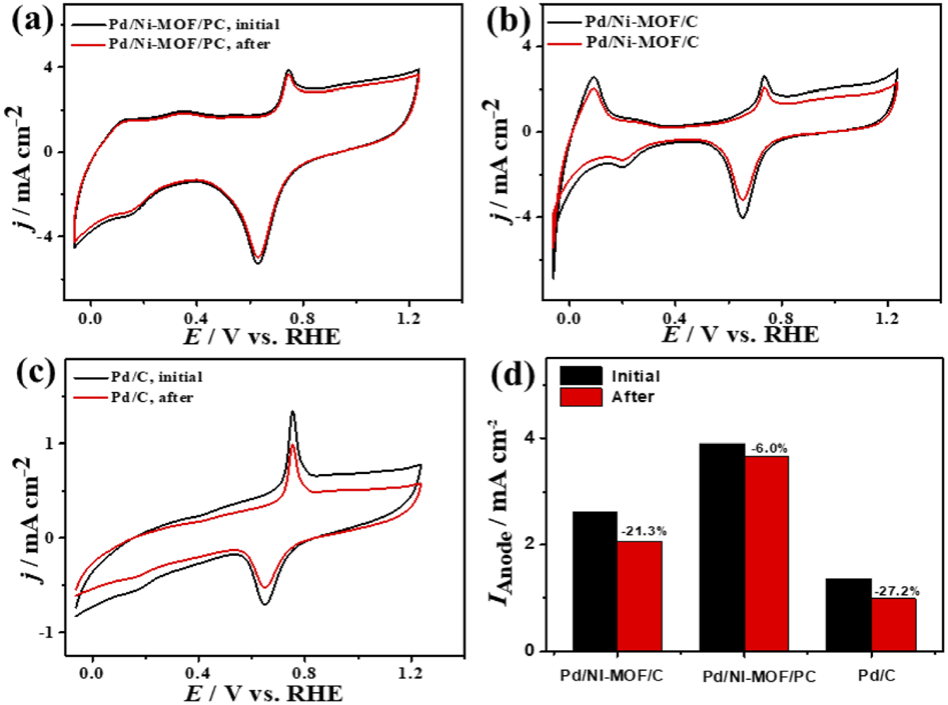
(a–c) CV of CO oxidation durability and (d) *I*_anode_ stability of Pd/Ni-MOF/PC, Pd/Ni-MOF/C, and Pd/C in CO-saturated 0.1 M KOH electrolyte.

**Fig. 7:**
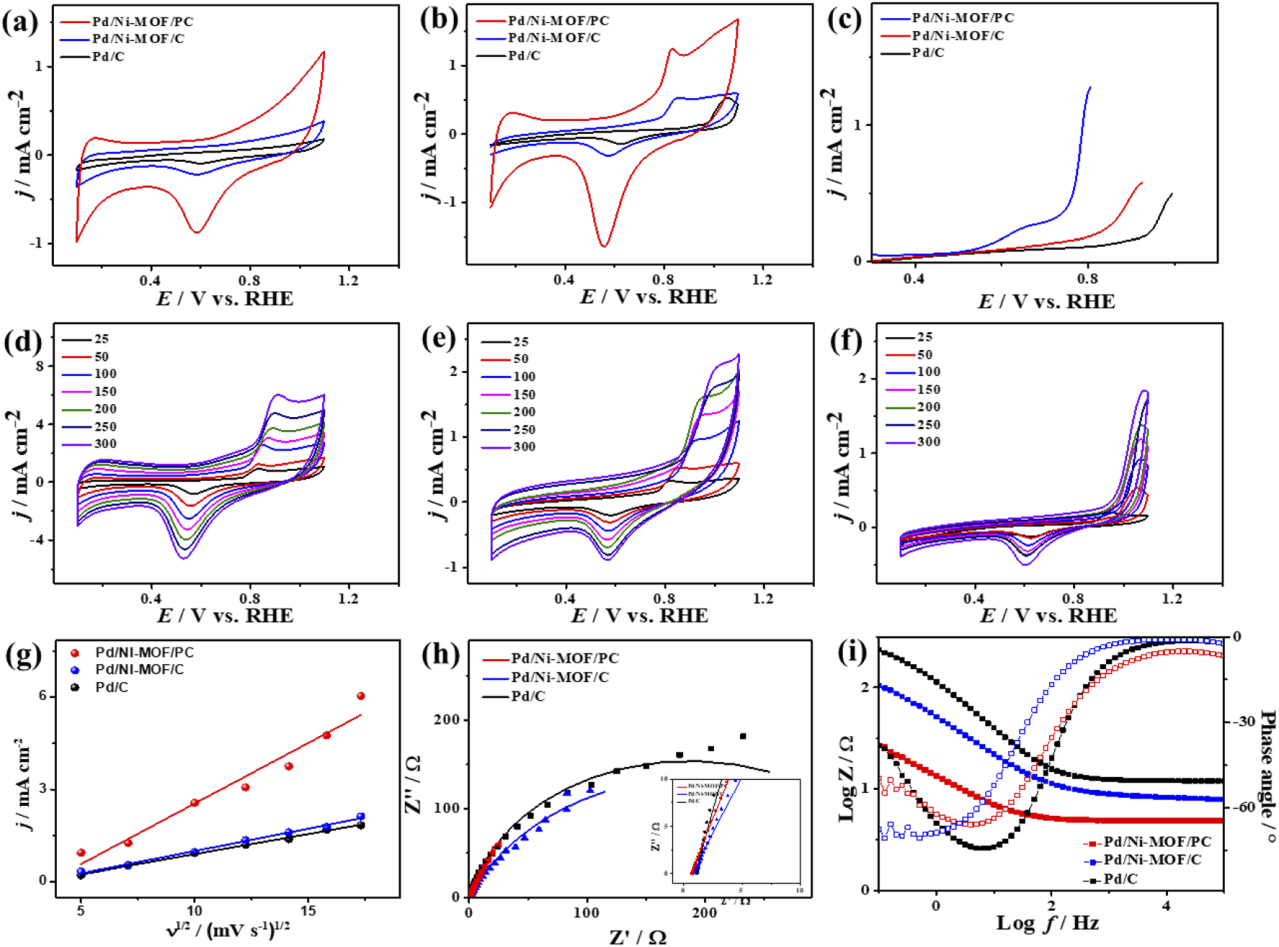
(a) CV in 0.1 M KOH, (b) CV in CO-saturated 0.1 M NaHCO_3_, and (c) LSV in CO-saturated 0.1 M NaHCO_3_ of Pd/Ni-MOF–PC, Pd/Ni-MOF–C, and commercial Pd/C. CV measured in 0.1 M NaHCO_3_ at different scan rates of (d) Pd/Ni-MOF/PC, (e) Pd/Ni-MOF/C, and (f) Pd/C and their (g) related plots of current density *vs.* square root of scan rates, (h) Nyquist plots, and (i) Bode plots measured in CO-saturated 0.1 M NaHCO_3_ electrolyte of Pd/Ni-MOF/PC, Pd/Ni-MOF/C and Pd/C.

The ideality factor (0.5 < *a* < 1) of the catalysts for modeling with CPE rather than *C*_dl_ confirms the porous and heterogenous nature of the electrode surface rather than an ideal smooth surface. The phase angle in the range of 60–80° for all the catalysts further corroborates that the CO oxidation in the KOH electrolyte occurs on a heterogenous porous surface of pseudocapacitive property rather than an ideal double layer. Intriguingly, the *I*_anode_ of Pd/Ni-MOF/PC (3.94 mA cm^−2^) is superior to that of previously reported PdAg/C^64^ and PtNi multicubes^65^ and other catalysts measured under similar conditions (Table S2†). The CA (Fig. S6b†) measured for 2000 s in CO-saturated 0.1 M KOH electrolyte designates the higher CO oxidation durability of Pd/Ni-MOF/PC than Pd/Ni-MOF/C and Pd/C, as shown in the inferior current degradation. Meanwhile, the CV tests measured after CA on all catalysts sustained the initial CO oxidation voltammogram features but with superior stability of Pd/Ni-MOF/PC. To this end, the *I*_anode_ of Pd/Ni-MOF/PC diminished only by 6.0% compared with Pd/Ni-MOF/C (21.3%) and Pd/C (27.2%) (Fig. 6a–d). The CVs measured in 0.1 M NaHCO_3_ demonstrate the voltammogram merits of Pd-based catalysts but with a higher HUPD of Pd/Ni-MOF/PC than Pd/Ni-MOF/C and Pd/C, inferring its higher ECSA (Fig. 7a). The ECSA of Pd/Ni-MOF/PC (14.75 m^2^ g^−1^) is 3.2 times that of Pd/Ni-MOF/C (4.55 m^2^ g^−1^) and 4.03 times that of Pd/C (3.66 m^2^ g^−1^), inferring the greater active sites of Pd/Ni-MOF/PC. The CV curves measured in CO-saturated NaHCO_3_ reveal the CO oxidation voltammogram with apparent *I*_anode_ and *I*_cathode_ in the potential, but with a significantly higher activity of Pd/Ni-MOF/PC (Fig. 7b). The *I*_anode_ of Pd/Ni-MOF/PC (1.26 mA cm^−2^) is greater than that of Pd/Ni-MOF/C (0.55 mA cm^−2^) by 2.29 times and Pd/C (0.53 mA cm^−2^) by 2.3 times, implying the maximized atomic utilization of Pd–Ni–N_*x*_ in Pd/Ni-MOF/PC, owing to its hierarchal porous structure. The LSV reveals the ability of Pd/Ni-MOF/PC to deliver higher *I*_anode_ under any applied potential than Pd/Ni-MOF/C and Pd/C, implying the quick CO oxidation kinetics on Pd/Ni-MOF/PC (Fig. 7c).

**Fig. 8:**
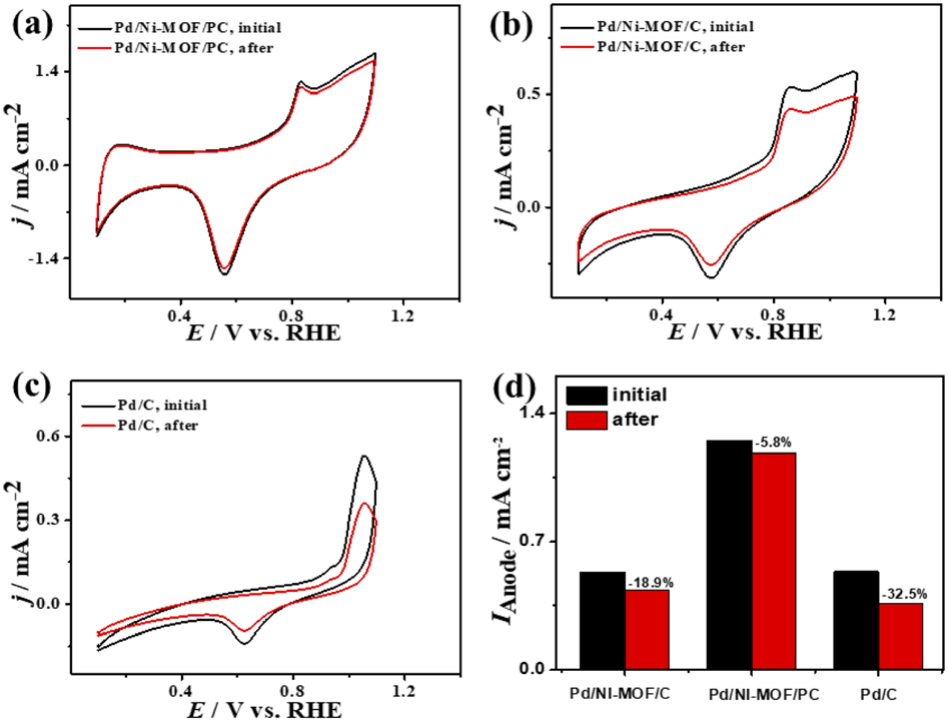
(a–c) CV stability of CO oxidation and (d) *I*_anode_ stability of Pd/Ni-MOF/PC, Pd/Ni-MOF/C, and Pd/C in CO-saturated 0.1 M NaHClO_4_ electrolyte.

That is additionally seen in the substantially lower *E*_onset_/*E*_oxi_ (Fig. S7a†) of Pd/Ni-MOF/PC (0.73 V/0.83 V) relative to Pd/Ni-MOF/C (0.75 V/0.85 V) and Pd/C (0.97 V/1.05 V). A similar CO oxidation activity trend was observed with LSV. The *I*_anode_ increased steadily with increasing *ν* from 25 to 300 mV s^−1^ on the as-synthesized catalysts but with a higher *I*_anode_ value on Pd/Ni-MOF/PC than Pd/Ni-MOF/C and Pd/C (Fig. 7d–f). The linear relationship among *I*_anode_*vs. ν*^1/2^ (Fig. 7g) suggests the diffusion-controlled process of CO oxidation. However, Pd/Ni-MOF/PC exposes a larger slope of (0.39) than Pd/Ni-MOF/C (0.15) and Pd/C (0.13), suggesting fast CO diffusion on the Pd/Ni-MOF/PC. The Nyquist plots of EIS measured on all electrocatalysts reveal a semicircle line but with a smaller diameter of Pd/Ni-MOF/PC than Pd/Ni-MOF/C and Pd/C (Fig. 7h), implying the better electrolyte–electrode interaction and quicker charge mobility on Pd/Ni-MOF/PC. That is seen in the lower *R*_s_ and *R*_ct_ besides a greater CPE on Pd/Ni-MOF/PC relative to Pd/Ni-MOF/C and Pd/C (Table 3). The ideality factor (0.5 < *a* < 1) of the catalysts for modeling with CPE rather than *C*_dl_ reveals that their CO oxidation is a diffusion-controlled process coupled to the adsorption process. The CAs (Fig. S7b†) measured for 1200 s in the CO-purged 0.1 M NaHCO_3_ electrolyte indicate the high durability of Pd/Ni-MOF/PC than Pd/Ni-MOF/C and Pd/C as appeared in the slower current attenuation. The CVs measured after the CA test in the CO-saturated NaHCO_3_ electrolyte (Fig. 8a–c) show that all catalysts earmarked their CO oxidation voltammogram features. Pd/Ni-MOF/PC kept 95% of its initial *I*_anode_ compared to Pd/Ni-MOF/C (90%) and Pd/C (80%), indicating the superior stability of Pd/Ni-MOF/PC (Fig. 8d). These results warrant that the oxidation performance of Pd/Ni-MOF/PC in HClO_4_ is superior to that in KOH and NaHCO_3_. This is plausibly due to the greater CO-adsorption ability in HClO_4_ relative to KOH and NaHCO_3_ as obtained from the integration of the anodic peak current (Table S2†). The significant enhancement of the CO oxidation performance of Pd/Ni-MOF/PC plausibly originated from the porous 2D morphology and Pd/Ni–N_*x*_ active sites.^38,43–47^

**Table 3:** EIS data for catalysts in CO-purged 0.1 M NaHCO_3_ electrolyte

	*R*_s_/Ω	*R*_ct_/Ω	CPE/μS s^−*a*^	*a*
Pd/C	1.15 ± 0.09	234.69 ± 3.37	25.74 ± 0.26	0.88
Pd/Ni-MOF/C	0.98 ± 0.04	102.89 ± 2.19	66.85 ± 0.68	0.77
Pd/Ni-MOF/PC	0.68 ± 0.03	26.42 ± 1.53	217.10 ± 2.15	0.82

Porous and structure defective MOF/PC is endowed with outstanding rich electron density, higher electrical conductivity, and enhanced charge transfer; meanwhile, multiple pores enhance the adsorption of reactant molecules (*i.e.*, CO and O_2_) and facilitate their diffusion to the inner cavities, which are highly stable against agglomeration.^38,43–47^ In addition, the abundant active sites of Pd/Ni–N_*x*_ embedded in MOF/PC allow the activation/dissociation of CO and O_2_ along with facilitating the generation of oxygen species (*i.e.*, OH) at low overpotentials. Exploring MOF/PC with Pd/Ni–N_*x*_ active sites tailors the electrolyte–electrode interaction and charge transfer resistance resulting in dissimilar CO oxidation activity and voltammogram features in different electrolytes.^66–68^

## Conclusion

This study presents the rational synthesis of Pd/Ni-MOF/PC *via* the microwave-irradiation and carbonization at 900 °C, followed by chemical etching. Pd/Ni-MOF/PC comprises porous 2D carbon nanosheets with abundant pores and Pd/Ni–N_*x*_ active sites originating from the *in situ* emitted gases from triethylamine that facilitate the reduction of Ni-impeded MOF to form Ni nanocrystals, while chemical etching allows the partial etching of Ni nanocrystals, which results in the formation of larger pores and more exposed active sites on the deposition of Pd to form Pd/Ni–N_*x*_. These merits endowed the CO oxidation activity substantially more than that of Pd/Ni-MOF/C (with low porosity or less active Pd/Ni–N_*x*_ sites formed without etching) and commercial Pt/C catalyst in HClO_4_, KOH, and NaHCO_3_ electrolytes. Using different electrolytes alters the CO oxidation activity and voltammogram features, but Pd/Ni-MOF/PC was superior to other catalysts, as shown in the high *I*_anode_, low *E*_oxi_, *E*_onset_, and impedance. The CO oxidation activity of Pd/Ni-MOF/PC was in the order of HClO_4_ > KOH > NaHCO_3_. This study may allow the fabrication of MOF/PC incorporated with various metals for electrochemical CO oxidation.

## Conflicts of interest

The authors declare no conflict of interest.

## Supplementary Material

NA-004-D2NA00455K-s001
