# Correction: Pd/Ni-metal–organic framework-derived porous carbon nanosheets for efficient CO oxidation over a wide pH range

**DOI:** 10.1039/d2na90092k

**Published:** 2022-12-02

**Authors:** Adewale K. Ipadeola, Kamel Eid, Aboubakr M. Abdullah, Rashid S. Al-Hajri, Kenneth I. Ozoemena

**Affiliations:** Center for Advanced Materials, Qatar University Doha 2713 Qatar bakr@qu.edu.qa; Gas Processing Center (GPC), College of Engineering, Qatar University Doha 2713 Qatar kamel.eid@qu.edu.qa; Petroleum and Chemical Engineering Department, Sultan Qaboos University Muscat Oman rashidh@squ.edu.om; Molecular Sciences Institute, School of Chemistry, University of the Witwatersrand Private Bag 3, PO Wits Johannesburg 2050 South Africa Kenneth.ozoemena@wits.ac.za

## Abstract

Correction for ‘Pd/Ni-metal–organic framework-derived porous carbon nanosheets for efficient CO oxidation over a wide pH range’ by Adewale K. Ipadeola *et al.*, *Nanoscale Adv.*, 2022, https://doi.org/10.1039/d2na00455k.

The authors regret that one of the funders of this work was omitted in the Acknowledgements section of the original publication. The correct acknowledgements are as follows:

This work was supported by the Qatar University High Impact Internal Grant (QUHI-CAM-22/23-550), Qatar National Research Fund (NPRP13S-0117-200095) and NRF/DSI/Wits SARChi Chair in Materials Electrochemistry and Energy Technologies (MEET) (UID No. 132739). The statements made herein are solely the responsibility of the authors. The authors are grateful to the Environmental Science Center (ESC), Qatar University, for the ICP-OES analysis. The publication of this article was funded by Qatar National Library.

The Royal Society of Chemistry apologises for these errors and any consequent inconvenience to authors and readers.

## Supplementary Material

