# Supplementary material for: Pd/Ni-metal–organic framework-derived porous carbon nanosheets for efficient CO oxidation over a wide pH range
Source: Nanoscale Adv. 2022 Sep 5;4(23):5044–55. doi: 10.1039/d2na00455k (PMC9680948; doi:10.1039/d2na00455k)
Supplement: NA-004-D2NA00455K-s001 [file NA-004-D2NA00455K-s001.pdf]

Electronic Supplementary Material (ESI) for Nanoscale Advances.
This journal is © The Royal Society of Chemistry 2022

Supplementary Information

Pd/Ni-Metal-organic Framework-derived Porous Carbon Nanosheets for Efficient CO Oxidation over a Wide pH Range

Adewale K. Ipadeola[a], Kamel Eid[b*], Aboubakr M. Abdullah[a*] Rashid S. Al-Hajri[c], and Kenneth I. Ozoemena[d*]

[a] Center for Advanced Materials, Qatar University, Doha 2713, Qatar. E-mail: bakr@qu.edu.qa

[b] Gas Processing Center(GPC), College of Engineering, Qatar University, Doha 2713, Qatar. E-mail: kamel.eid@qu.edu.qa

[d] Petroleum and Chemical Engineering Department, Sultan Qaboos University, Muscat, Oman

[d] Molecular Sciences Institute, School of Chemistry, University of the Witwatersrand, Private Bag 3, PO Wits, Johannesburg 2050, South Africa. E-mail: Kenneth.ozoemena@wits.ac.za

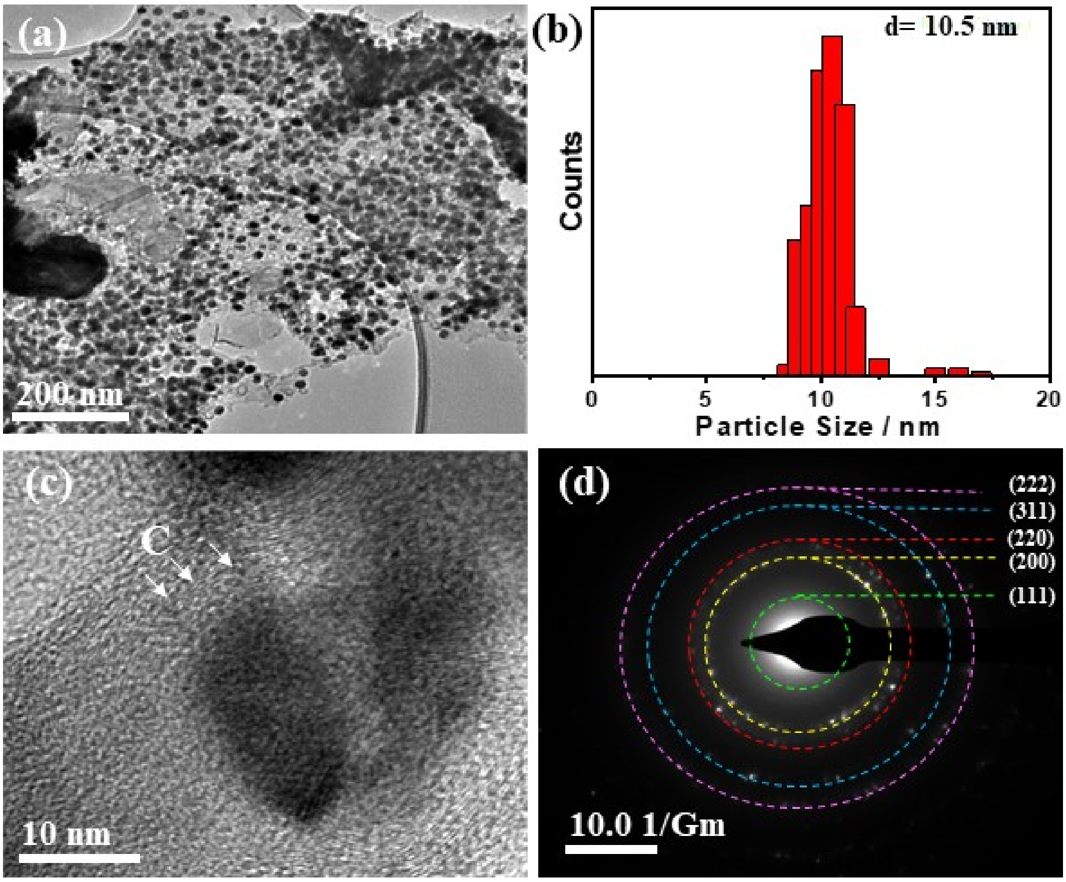

Figure S1. (a) TEM image, (b) particle size distribution of Pd nanoparticles, (c) HRTEM image and (d) SAED of Pd/Ni-MOF/C

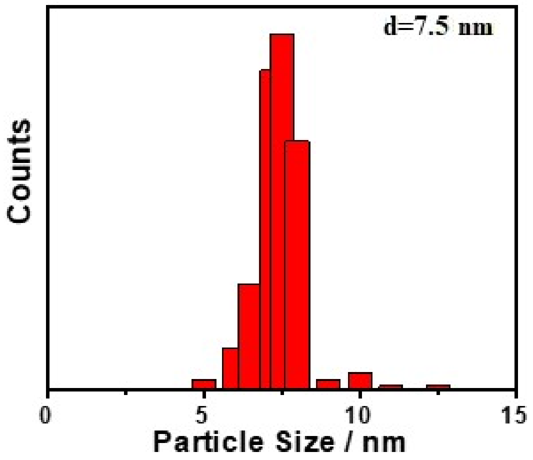

Figure S2. Particle size distribution of Pd nanoparticles in Pd/Ni-MOF/PC

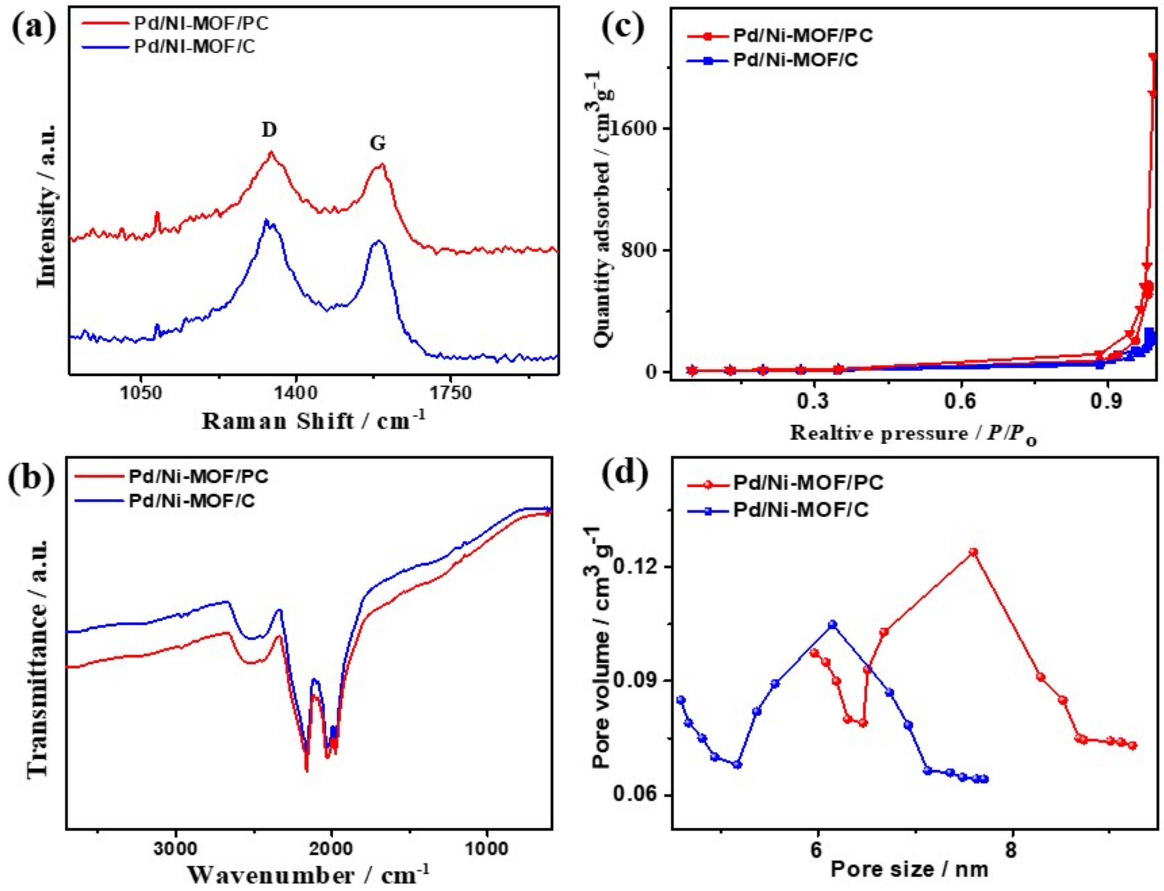

Figure S3. (a) Raman spectra, (b) FTIR spectra and (c,d) BET surface analysis of Pd/Ni-MOF/PC and Pd/Ni-MOF/C

Table S1: ICP-OES and BET analysis of Pd/Ni-MOF/PC and Pd/Ni-MOF/C

Elemental composition/ BET data	Pd/Ni-MOF/PC	Pd/Ni-MOF/C
Pd (wt. %)	14.26 ± 0.25	21.40 ± 1.05
Ni (wt. %)	14.94 ± 0.69	43.12 ± 0.91
Pore size / nm	7.60	6.15
Pore volume / cm^3/g	0.1240	0.1049
BET Surface area / m^2/g	153.0463	142.2231

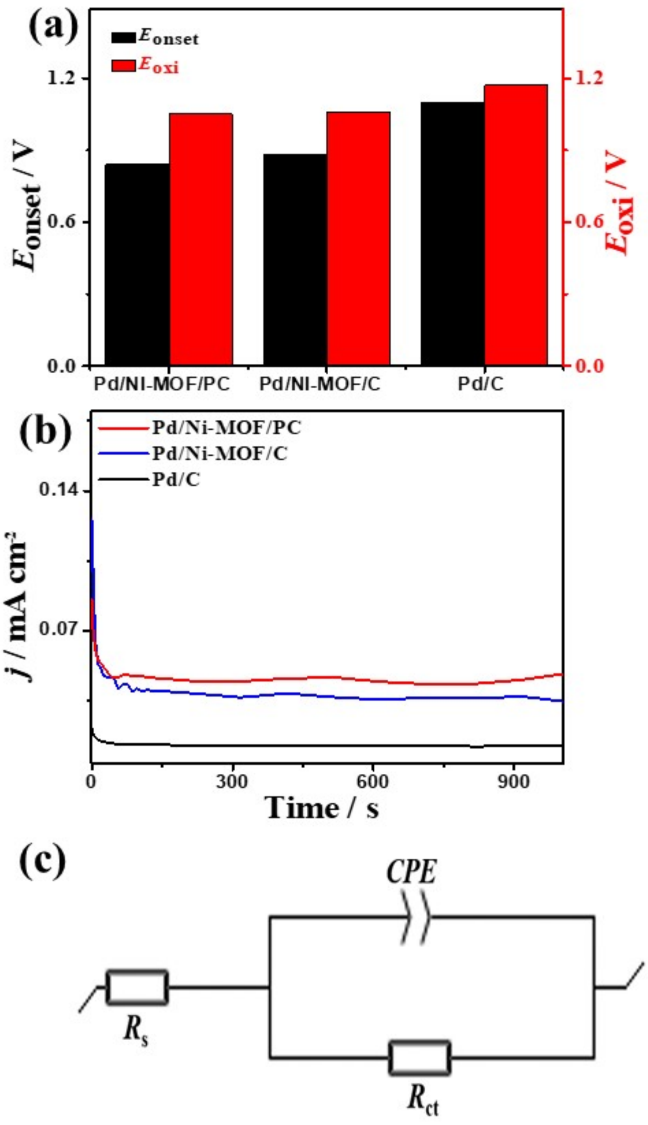

Figure S4. (a) Bar chart of E_{onset} and E_{oxi}, (b) Chronoamperometry in CO-saturated 0.1 M $HClO_4$, and (c) Voigt electrical equivalent circuit (EEC)

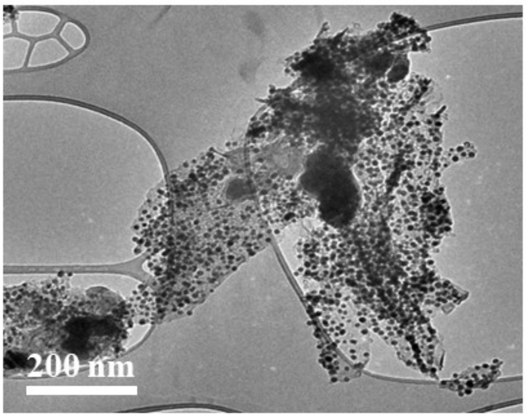

Figure S5. TEM of Pd/Ni-MOF/PC after stability

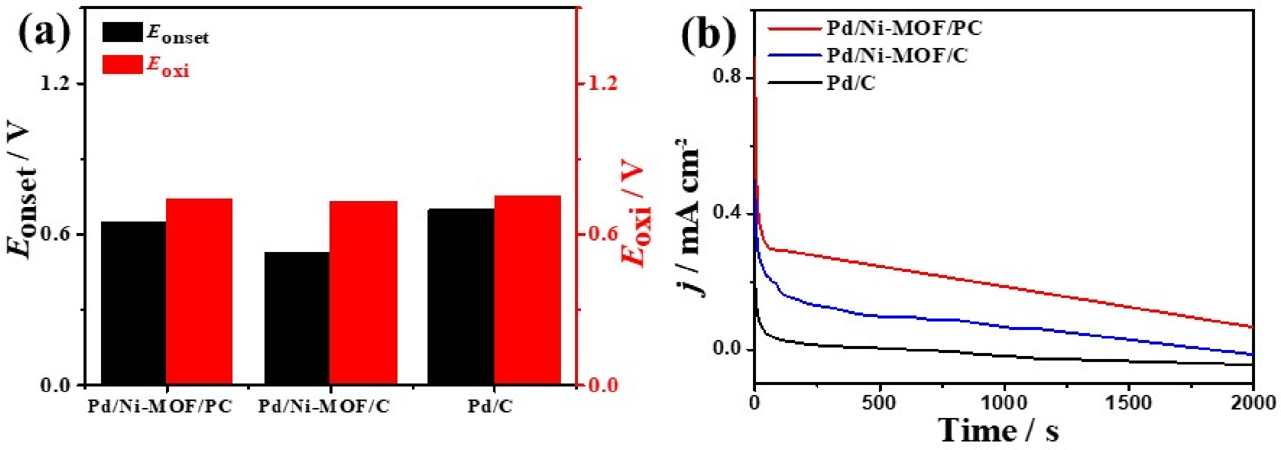

Figure S6. (a) Bar chart of E_{onset} and E_{oxi} and (b) Chronoamperometry in CO-saturated 0.1 M KOH

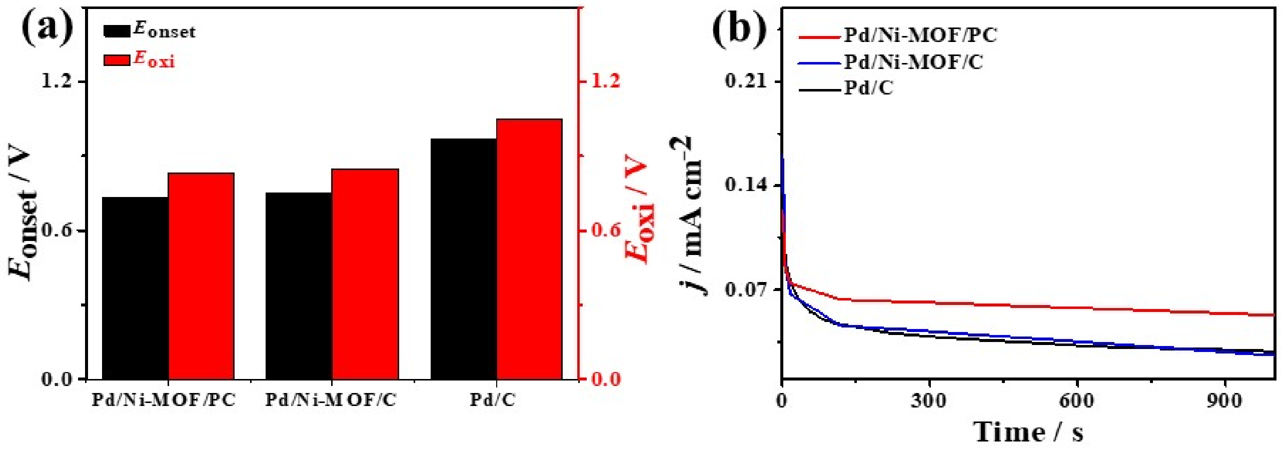

Figure S7. (a) Bar chart of E_{onset} and E_{oxi} and (b) Chronoamperometry in CO-saturated 0.1 M $NaHCO_3$

Table S2: Comparative CO oxidation of the electrocatalysts with literature. [$]mA, Scan rates (v), Reference electrodes (REs), Maximum current (I_{Anode}), and oxidation potential (E_{Oxi})

Electrocatalysts	Medium / v (mV/s) / REs	I_{Anode} (mA/cm^2) / E_{Oxi} (V)	Refs.
Pt(110)–Ru	0.5 M H_2SO_4 / 100 / RHE	[$]0.025 / 0.50	[1]
Pt-NbOx	0.5 M H_2SO_4 / 20 / RHE	0.500 / 0.75	[2]
Well-ordered Pt(111)	0.1 M NaOH / 50 / RHE	0.500 / 0.80	[3]
PtRu (1:1)	0.1 M $HClO_4$ / 50 / Ag/AgCl	0.120 / 0.25	[4]
Pt/SnO_x	1 M $HClO_4$ / 20 / RHE	0.870 / 0.70	[5]
Pd/$Ti_3C_2T_x$	0.1 M $HClO_4$ / 50 / Ag/AgCl	0.31/ 0.9	[6]
Pt(FAM)	0.1 M H_2SO_4 / 50 / RHE	0.320 / 0.72	[7]
Dendrimer-encapsulated Pt nanoparticles	0.1 M $HClO_4$ / 50 / Hg/Hg_2SO_4	0.200 / 0.30	[8]
Polycrystalline Pd	0.5 M H_2SO_4 / 20 / RHE	0.175 / 0.90	[9]
PdAg/C	0.5 KOH/ 20 / RHE	0.944 / 0.60	[10]
PtPd nanodendrites	1.0 M KOH / 50 / Ag/AgCl	5.100 / -0.15	[11]
60 wt. % Pt/C	0.5 H_2SO_4 / 10 / SHE	0.200 / 0.64	[12]
PtRu@h-BN/C	0.1 M H_2SO_4 / 20 / RHE	1.250 / 0.60	[13]
PtNi multicubes	1 M KOH / 50 / RHE	0.580 / 0.65	[14]
Pt polyhedron with smooth surfaces	0.5 M H_2SO_4 / 50 / RHE	0.300 / 0.80	[15]
Pd/Ni-MOF/PC	0.1 M $HClO_4$ / 50 / RHE	4.701 / 1.05	This work
	0.1 M KOH / 50 / RHE	3.936 / 0.74	
	0.1 M $NaHCO_3$ / 50 / RHE	1.220 / 0.83	
Pd/Ni-MOF/C	0.1 M $HClO_4$ / 50 / RHE	1.356 / 1.06	This work
	0.1M KOH / 50 / RHE	2.660 / 0.73	
	0.1 M $NaHCO_3$ / 50 / RHE	0.526 / 0.85	

References

1. J. C. Davies, B. E. Hayden and D. J. Pegg, *Electrochim. acta*, 1998, **44**, 1181-1190.
2. A. Ueda, Y. Yamada, T. Ioroi, N. Fujiwara, K. Yasuda, Y. Miyazaki and T. Kobayashi, *Catal. Todays*, 2003, **84**, 223-229.
3. J. Spendelow, J. Goodpaster, P. J. A. Kenis and A. Wieckowski, *J. Phys. Chem.*, 2006, **110**, 9545-9555.
4. B. Du, S. A. Rabb, C. Zangmeister and Y. Tong, *Phys. Chem. Chem. Phys.*, 2009, **11**, 8231-8239.
5. T. Matsui, K. Fujiwara, T. Okanishi, R. Kikuchi, T. Takeguchi and K. Eguchi, *J. Power Sources*, 2006, **155**, 152-156.
6. B. Salah, K. Eid, A. M. Abdelgwad, Y. Ibrahim, A. M. Abdullah, M. K. Hassan and K. I. Ozoemena, *Electroanalysis*, 2022, **34**, 677-683.
7. E. G. Ciapina, S. F. Santos and E. R. Gonzalez, *J. Electroanal. Chem.*, 2010, **644**, 132-143.
8. M. G. Weir, V. S. Myers, A. I. Frenkel and R. M. J. C. Crooks, *ChemPhysChem*, 2010, **11**, 2942-2950.
9. L.-l. Fang, Q. Tao, M.-f. Li, L.-w. Liao, D. Chen and Y.-x. Chen, *Chinese J. Chem. Phys.*, 2010, **23**, 543-548.
10. T. Jurzinsky, C. Cremers, K. Pinkwart and J. Tübke, *Electrochim. Acta*, 2016, **199**, 270-279.
11. K. Eid, Y. H. Ahmad, H. Yu, Y. Li, X. Li, S. Y. AlQaradawi, H. Wang and L. J. N. Wang, *Nanoscale*, 2017, **9**, 18881-18889.
12. I. J. McPherson, P. A. Ash, L. Jones, A. Varambhia, R. M. Jacobs and K. A. Vincent, *J. Phys. Chem. C*, 2017, **121**, 17176-17187.
13. M. Sun, Y. Lv, Y. Song, H. Wu, G. Wang, H. Zhang, M. Chen, Q. Fu and X. Bao, *Appl. Surf. Sci.*, 2018, **450**, 244-250.
14. F. Wu, K. Eid, A. M. Abdullah, W. Niu, C. Wang, Y. Lan, A. A. Elzatahry and G. Xu, *ACS Appl. Mater. Interfaces*, 2020, **12**, 31309-31318.
15. D. Shen, Y. Liu, G. Yang, H. Yu, P.-F. Liu and F. Peng, *Appl. Catal. B.l*, 2021, **281**, 119522.
